# Efficacy of Different Sterilization Techniques for Toothbrush Decontamination: An Ex Vivo Study

**DOI:** 10.7759/cureus.21117

**Published:** 2022-01-11

**Authors:** Ahmad S Assari, Mohammed Mohammed Mahrous, Yahia A Ahmad, Faisal Alotaibi, Muath Alshammari, Firas AlTurki, Thwaini AlShammari

**Affiliations:** 1 Oral and Maxillofacial Surgery and Diagnostic Sciences, Riyadh Elm University, Riyadh, SAU; 2 Restorative Dentistry, Riyadh Elm University, Riyadh, SAU; 3 Dentistry, Riyadh Elm University, Riyadh, SAU

**Keywords:** chlorhexidine gluconate, glutaraldehyde, hydrogen peroxide, listerine, povidone-iodine, sodium hypochlorite, toothbrush contamination, toothbrush sterilization, uv brush sterilization, white alcohol

## Abstract

Background

Contaminated toothbrushes can cause several oral and systemic illnesses. This study aimed to identify the most rapid, effective, and affordable method for toothbrush decontamination. In addition, the most prevalent bacterial species colonizing toothbrushes were determined.

Methodology

Toothbrushes were collected after two weeks of use by 55 volunteers. The bacterial count was measured before and after sterilization using 0.2% chlorhexidine gluconate, 0.1% Listerine, 70% white alcohol, 10% povidone-iodine, 1% sodium hypochlorite, 2% glutaraldehyde, ultraviolet radiation, microwave irradiation, 3% hydrogen peroxide, and 100% white vinegar, with tap water rinse as the control.

Results

A marked reduction in the bacterial count was observed pre- and post-sterilization. All sterilization methods were effective for toothbrush disinfection. Sterilization using 2% glutaraldehyde and 3% hydrogen peroxide solutions resulted in the most significant reduction in the mean bacterial count and percentage reduction in the total bacterial count, respectively. The toothbrush samples were also colonized by several different types of bacteria. The most common colonizing bacterial species included *Bacillus subtilis *(28% prevalence),* Sacrina *(26% prevalence),and* Streptococcus pneumoniae *(24% prevalence).

Conclusions

Because bacterial contamination cannot be eliminated and different species colonize toothbrush surfaces, cleaning and disinfection are essential to prevent disease transmission.

## Introduction

Toothbrushes used at home contain many bacteria that can contaminate the oral cavity [1]. These microorganisms can remain on the toothbrush for days or weeks after brushing [2]. Studies have reported a significant relationship between tooth decay and bacterial contamination of toothbrushes [3]. In general, after brushing, the toothbrush is rinsed with running water and stored. Hence, the level of bacterial contamination on a toothbrush depends on whether it is rinsed after brushing [4].

Contaminated toothbrushes may cause oral and systemic illnesses, including sepsis, gastrointestinal disorders, cardiovascular disease, respiratory distress, and kidney disease [5]. Brushing the teeth with a toothbrush for 30 seconds and up to four minutes can contaminate the toothbrush with bacteria, viruses, and fungi present in the oral cavity [6]. The oral cavity contains the highest number of bacteria among all other body areas [7]. Moreover, toothbrushes contain 4 × 108 colony-forming units (CFU)/mL of bacteria, mainly comprising the following species: *Staphylococci *(64%), *Escherichia *(57%), and *Pseudomonas *(28%). Due to storage conditions or poor hand hygiene, toothbrushes can be contaminated with microbes present in the air. In addition, toothbrushes are usually stored in the toilet and are heavily contaminated by intestinal bacteria in the air [8]. Therefore, toothbrush disinfection is essential to maintain toothbrush and oral hygiene [9].

Furthermore, toothbrush disinfection is necessary to prevent the spread of diseases, particularly in children, older adults, and high-risk patients, including those with immunodeficiency or those receiving organ transplants or chemotherapy [5]. Reducing toothbrush contamination may also be effective against various oral diseases [10]. Several studies on toothbrush disinfection are underway, including those using an ultraviolet (UV) toothbrush sterilizer, immersion disinfection, antibacterial solution sprays, microwave ovens, and dishwashers. Overall, toothbrush disinfection should be quick, effective, cheap, non-toxic, and easy to perform [10].

The use of chemical reagents remains an effective and convenient method for toothbrush disinfection. A previous study noted that on brushing teeth after using an antibacterial mouthwash, toothbrushes showed no bacteria. Bacteria in the mouth were significantly reduced after immersing a toothbrush in Listerine mouthwash for 20 minutes [8]. Several pediatric studies have reported the effectiveness of disinfecting toothbrushes with other solutions containing 0.12% chlorhexidine, such as mouthwashes and sprays [11]. Another laboratory experiment found no statistical difference between chlorhexidine-coated filament toothbrushes compared to the control group without coating [12].

These previous studies used various disinfection methods; however, none of the methods have been demonstrated to be cost-effective. Therefore, this study aimed to identify the most prevalent bacterial species on the bristle head of toothbrushes. In addition, it compared the efficiency of various liquid sterilization methods and UV and microwave irradiation on toothbrush decontamination.

## Materials and methods

Study volunteers

This study included 55 healthy volunteers aged 18-70 years from Riyadh, Saudi Arabia. Demographic data, medical history, oral hygiene practices, and oral checkup details were collected from all volunteers. To be included in the study sample, patients had to fulfill the following criteria: having at least three single-rooted and two multi-rooted functional teeth per quadrant (excluding third molars), free from systemic diseases, and decayed, missing, or filled teeth score of <3. We excluded the following patients: those using antibiotics or antiseptic mouthwashes for at least three months prior to the study and at the time of the study; undergoing any dental treatment, orthodontic treatment, or with an extensive intraoral prosthesis; using triclosan, chlorhexidine, cetylpyridinium chloride, and fluoride in any form as an oral hygiene aid; and those under the influence of drugs and tobacco.

The study was approved by the Institutional Review Board Committee at Riyadh Elm University (FUGRP/2021/232/424/418), and written informed consent was obtained from all participants.

Pre-study protocol

An oral hygiene kit with a toothbrush and paste was provided to all study volunteers. The bristles of all toothbrushes were of the same type to ensure uniform mechanical plaque control. The fluoridated dentifrice included in the kit (80 g) helped improve adherence to toothbrushing and maintain consistency in plaque control measures among the volunteers.

The volunteers brushed their teeth twice a day (approximately 1 g of toothpaste in the morning and evening) for two to four minutes using the provided toothpaste for more than two weeks. After brushing, the toothbrush was rinsed with running tap water for 30 seconds and placed in an open brush holder outside the bathroom with the bristles facing up without coverage.

Sample collection

**Figure 1:**
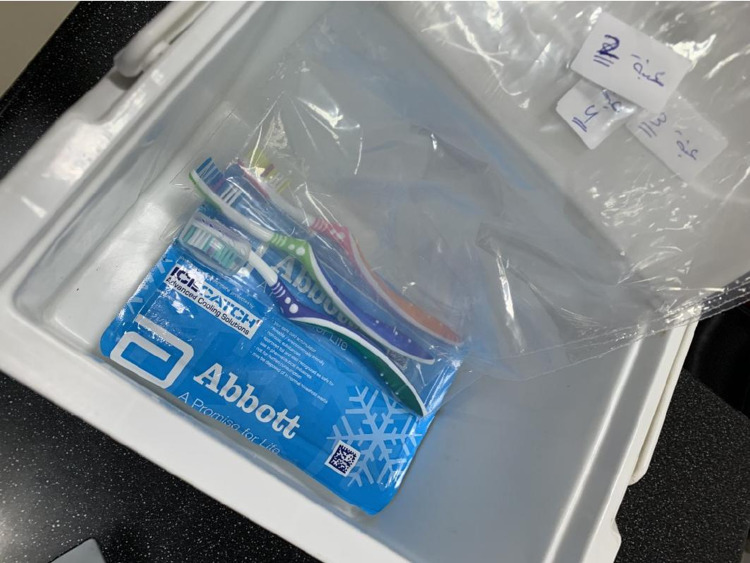
Toothbrush samples collected in sterilized plastic bags.

The toothbrushes were collected, placed in sterile plastic bags after the volunteers used them for two weeks (Figure 1), labeled (from 1 to 55), and immediately sent to the laboratory for evaluation. The brush samples were first rinsed with tap water and then analyzed.

Pre-sterilization microbial count

**Figure 2:**
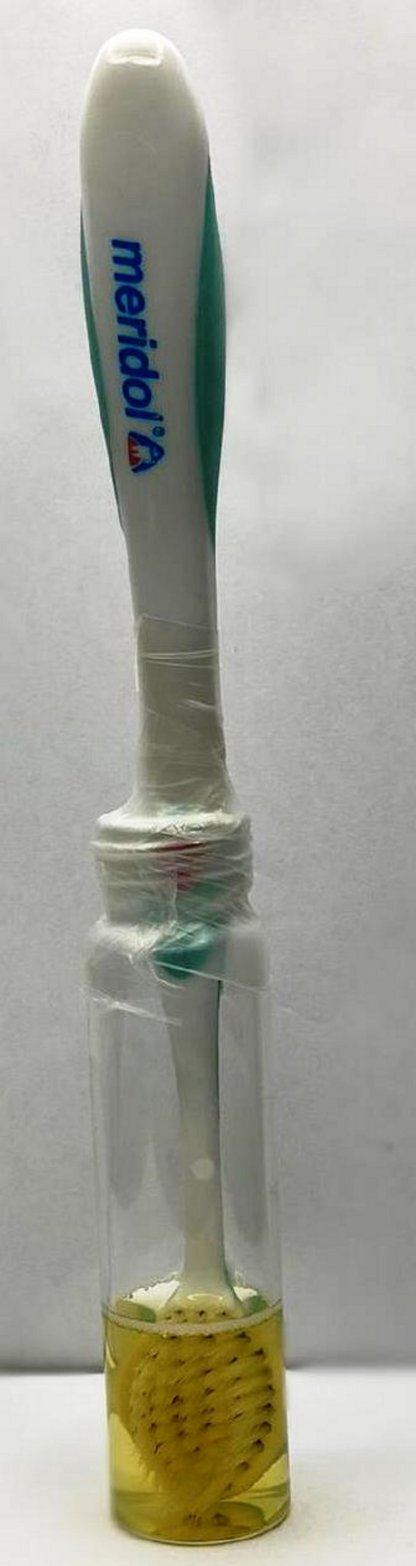
A sample toothbrush dipped in nutrient agar broth after collection from the volunteers.

The toothbrush samples were dipped in freshly prepared, sterile nutrient agar broth tubes (Figure 2). Each coded toothbrush was dipped into the corresponding broth tube stored in an incubator at 37°C for 48 hours to ensure the best possible retrieval of microbial species. After incubation, the nutrient broth tubes were vortexed for one minute. Approximately 0.1 mL of broth from each sample was dispensed on nutrient and blood agar plates. The agar plates were stored in an incubator at 37°C for 48 hours and examined for bacterial colonies.

**Figure 3:**
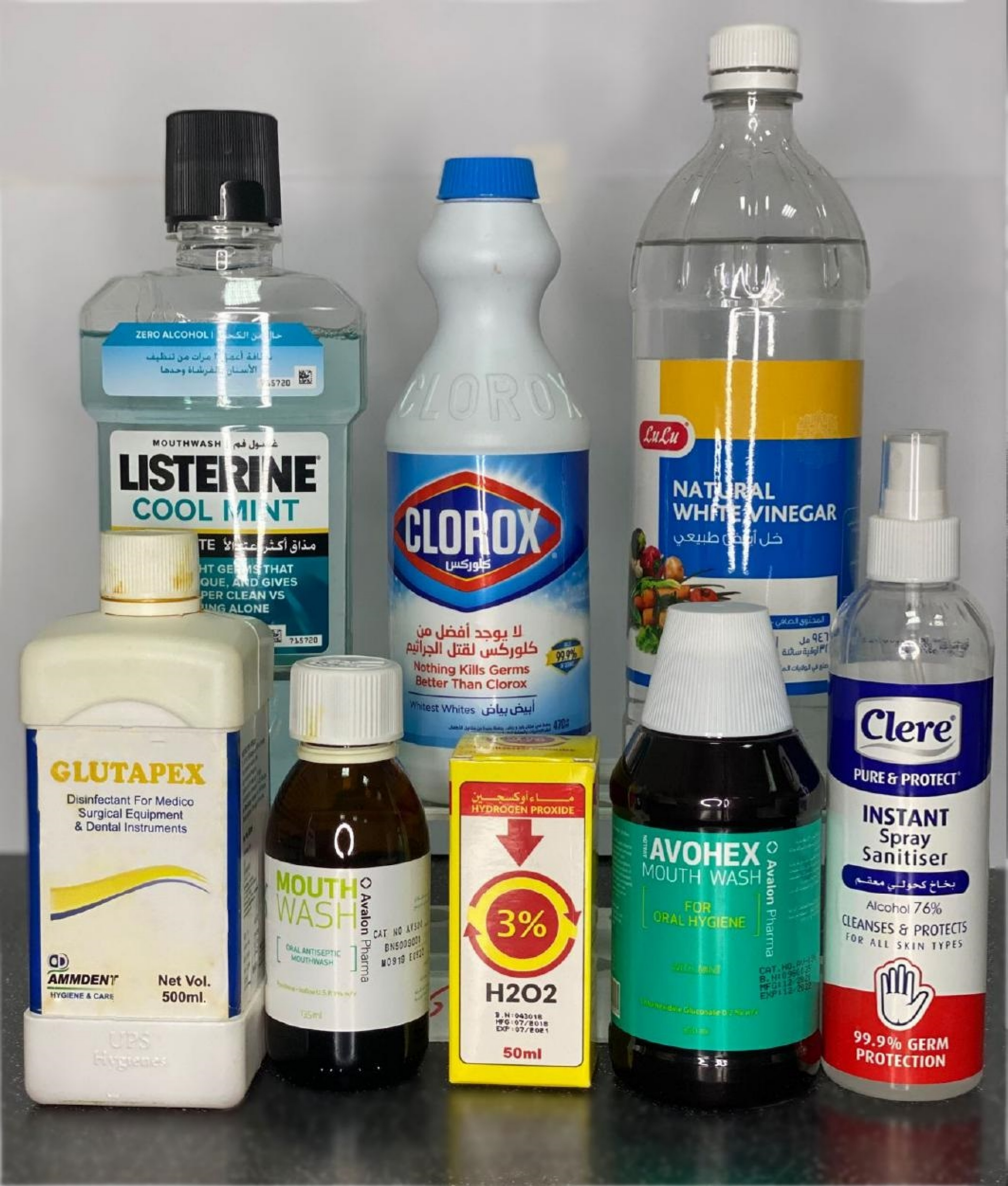
Various chemicals used for toothbrush sterilization.

**Figure 4:**
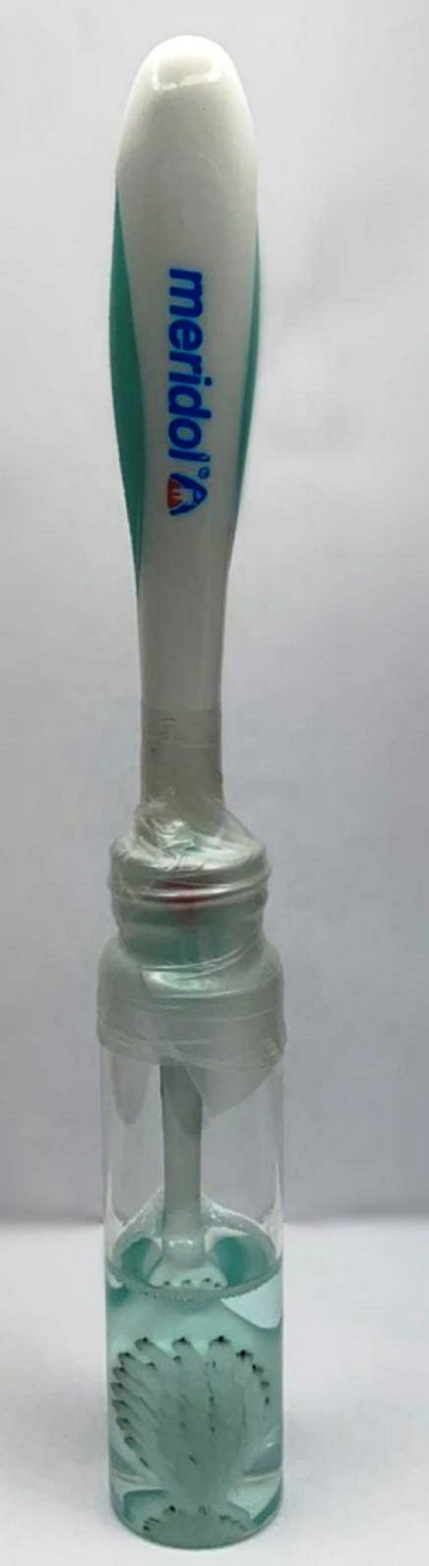
A sample toothbrush soaked in 10% povidone-iodine solution.

**Figure 5:**
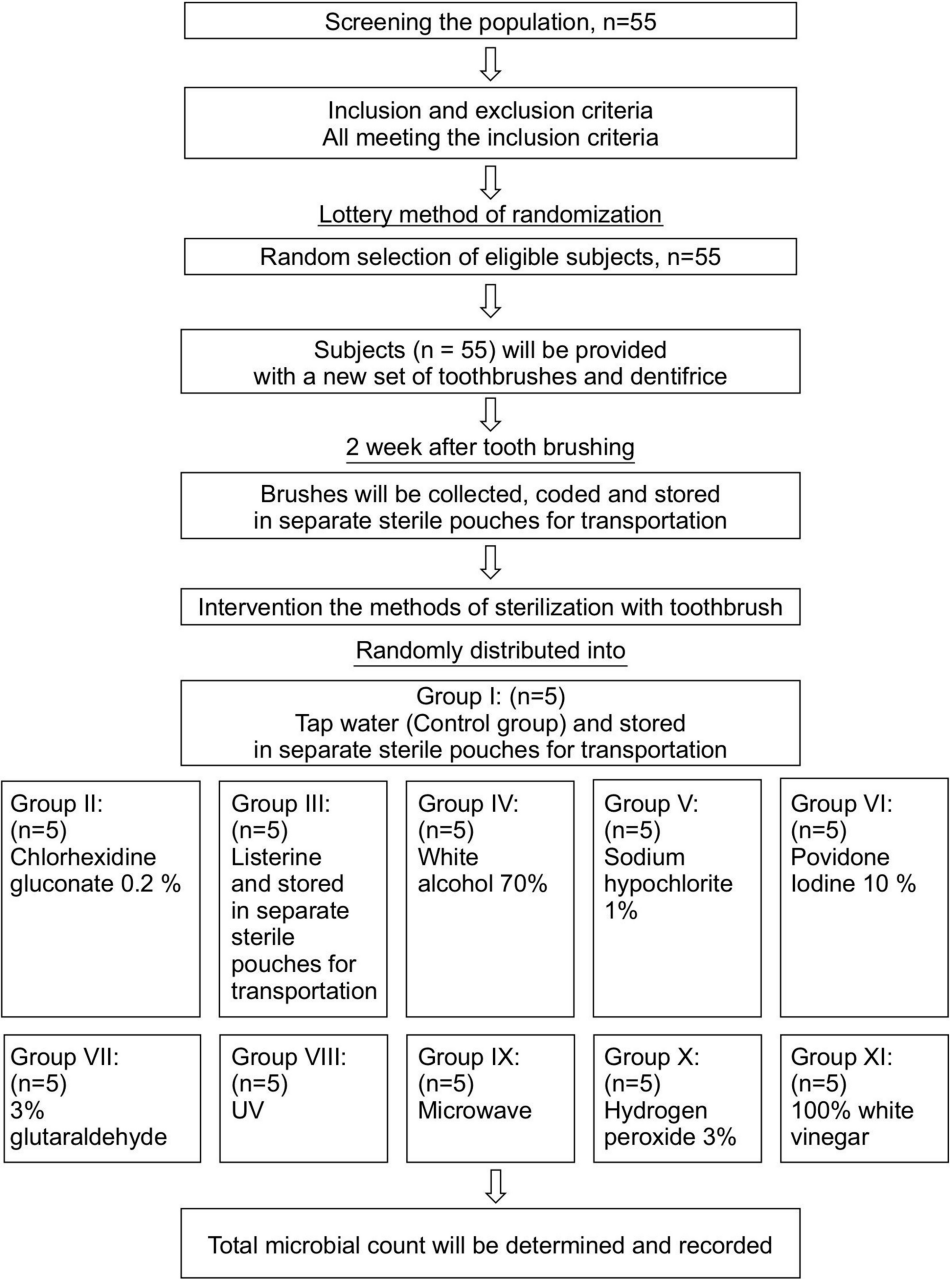
A summary of the study protocol.

After pre-sterilization counting of bacteria, the toothbrush samples were rinsed with tap water and innerved with various sterilization protocols. For sterilization, the toothbrush samples were randomly categorized into 11 groups (I-XI). Five toothbrush samples were allocated to each group. The details of the disinfection method employed for each group are listed in Table 1. Figure 3 shows the different types of disinfectants used. The toothbrushes were soaked in each disinfected solution (Figure 4). Figure 5 presents a summary of the study protocol.

**Table 1:** Random allocation of toothbrush samples to various methods of sterilization.

Groups	Sterilization methods	Sample number
Group I	Tap water	1–5
Group II	Chlorhexidine 0.2%	6–10
Group III	Listerine 0.1%	11–15
Group IV	White alcohol 70%	16–20
Group V	Sodium hypochlorite 1%	21–25
Group VI	Povidone-iodine 10%	26–30
Group VII	Glutaraldehyde 3%	31–35
Group VIII	Ultraviolet brush sterilizer	36–40
Group IX	Microwave	41–45
Group X	Hydrogen peroxide 3%	46–50
Group XI	White vinegar 100%	51–55

Post-sterilization microbial count

The toothbrush handles were sterilized using sterilizers for surgical instruments, placed in sterile nutrient solution tubes, and shaken vigorously for five minutes. Each sample was serially diluted from 10:1 to 10:12. The diluted solutions were spread evenly on nutrient agar and blood agar medium using the pouring cup method. Petri dishes were then incubated at 37°C for 48 hours to stimulate the formation of microbial colonies. A digital colony counter (Systonic, Panchkula, Haryana, India) was used to count the number of colonies as CFUs.

Identification of the most prevalent microbial species

The predominant colonies were identified and subcultured. The microorganisms were identified based on colony morphology, Gram staining, and biochemical testing, including catalase, oxidase, coagulase, and lactose fermentation tests.

Statistical analysis

SPSS software version 20 (IBM Corp., Armonk, NY, USA) was used for statistical analysis. One-way analysis of variance was used to compare and analyze the total bacterial count (CFU) after brushing for various groups. The number of bacteria before and after the procedure was compared using a paired t-test. Statistical significance was set at p-values of <0.05.

## Results

Mean bacterial CFU following toothbrush sterilization

We observed a significant reduction in the bacterial count (P < 0.05) following toothbrush sterilization by various methods. All sterilization methods were effective in decontaminating toothbrushes. Significant differences in bacterial counts among the various sterilization methods were noted (P < 0.05), indicating effectiveness (Table 2).

**Table 2:** Bacterial count (CFU) before and after sterilization of toothbrushes. CFU: colony-forming unit; SD: standard deviation

Sterilization methods	Before sterilization (mean ± SD)	After sterilization (mean ± SD)	P-value
Group I (tap water)	2,273 ± 1,249.61	1,868 ± 1,225.96	0.003
Group II (0.2% chlorhexidine gluconate)	3,717 ± 348.96	1,544 ± 399.47	0.000
Group III (0.1% Listerine)	3,143 ± 1,987.04	2,160 ± 1,214.26	0.029
Group IV (70% white alcohol)	3,261 ± 1,397.78	1,532 ± 1,162.87	0.000
Group V (1% sodium hypochlorite)	2,449 ± 1,013.42	1,834 ± 907.98	0.000
Group VI (10% povidine-iodine)	2,928 ± 538.91	778 ± 228.30	0.000
Group VII (2% gluteraldehyde)	1,524 ± 892.88	150 ± 90.32	0.000
Group VIII (ultraviolet sterilizer)	2,694 ± 961.26	622 ± 391.11	0.000
Group IX (microwave oven)	2,970 ± 490.05	1,280 ± 195.83	0.000
Group X (3% hydrogen peroxide)	2,006 ± 1,064.25	260 ± 176.49	0.006
Group XI (100% white vinegar)	2,306 ± 1,590	1,630 ± 1,094.28	0.002
P-value	0.004	0.000	-

Mean difference in reduction of bacterial count

The reduction in the bacterial count after sterilization was higher in groups VII and X, which involved using 2% glutaraldehyde (90.16% reduction) and 3% hydrogen peroxide (87.03% reduction), respectively. UV sterilizer and 10% povidone-iodine (PVI) solution resulted in 76.91% and 73.43% reduction in bacterial colonies, respectively. Other sterilization methods showed a marked reduction in bacterial colonies (Table 3).

**Table 3:** Comparison of mean difference and percentage reduction (between before and after sterilization) in bacterial count (CFU). CFU: colony-forming unit

Sterilization methods	Mean difference	Percentage reduction
Group I (tap water)	405	17.82%
Group II (0.2% chlorhexidine gluconate)	2,173	58.46%
Group III (0.1% Listerine)	983	31.28%
Group IV (70% white alcohol)	1,729	53.02%
Group V (1% sodium hypochlorite)	615	25.11%
Group VI (10% povidine-iodine)	2,150	73.43%
Group VII (2% gluteraldehyde)	1,374	90.16%
Group VIII (ultraviolet sterilizer)	2,072	76.91%
Group IX (Microwave oven)	1,690	56.90%
Group X (3% hydrogen peroxide)	1,746	87.03%
Group XI (100% white vinegar)	676	29.31%

Prevalence of various bacterial species from contaminated toothbrushes

**Figure 6:**
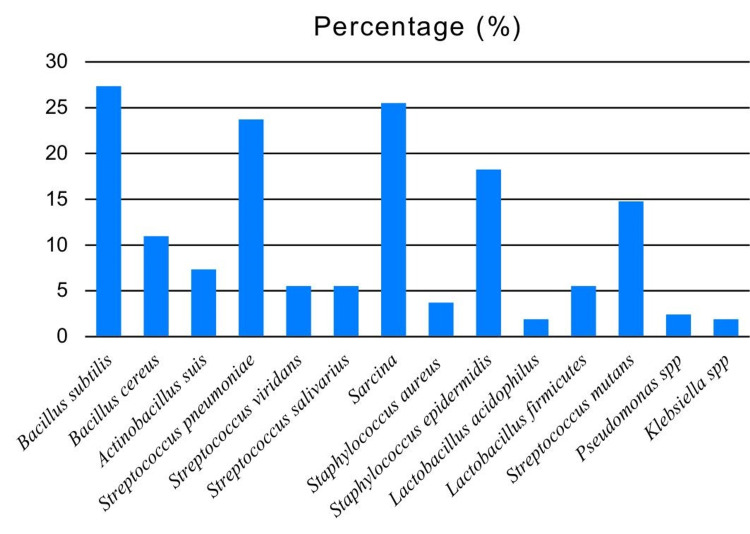
Prevalence of various bacterial species detected on used toothbrush samples.

**Figure 7:**
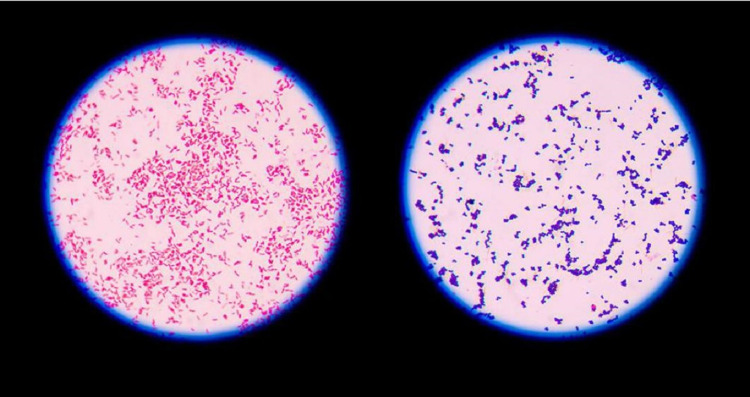
Identification of microbial species by Gram staining. Left: Gram-negative bacteria; right: Gram-positive bacteria.

We detected the following species in toothbrush samples: *Bacillus subtilis*, *B. cereus*, *Actinobacillus suis*, *Streptococcus pneumoniae*, *S. viridans*, *S. mutans*, *S. salivarius*, *Sarcina* spp., *Staphylococcus aureus*, *S. epidermidis*, *Lactobacillus acidophilus*, *L. firmicutes*, *Pseudomonas* spp., and *Klebsiella* spp. Of the 55 toothbrush samples, >25% showed *B. subtilis*, which was the most common bacterial isolate. The prevalence of other bacterial species is presented in Figure 6. Figure 7 shows a representative image for identifying Gram-negative and Gram-positive bacteria.

## Discussion

Tomar et al. investigated three disinfection methods, namely, chlorhexidine, UV, and saline sterilization, and reported a reduction in the average bacterial count after using different dilutions of the solutions and varying UV light intensities [11]. The difference in the bacterial count pre- and post-disinfection was statistically significant, suggesting that a contaminated toothbrush requires a particular disinfection procedure. da Silva et al. reported the effectiveness of submerging toothbrushes in 0.2% chlorhexidine for 20 minutes a day for disinfection [13]. Other studies reported that 100% of the bacterial population was removed from a toothbrush when soaked in 0.12% chlorhexidine solution [14] for two hours, followed by 0.2% chlorhexidine [15] for 20 hours. In this study, the reduction in the bacterial count after sterilization was higher in groups VII and X, which involved using 2% glutaraldehyde (90.16% reduction) and 3% hydrogen peroxide (87.03% reduction), respectively. UV sterilizer and 10% PVI solution resulted in 76.91% and 73.43% reduction in bacterial colonies, respectively. Amirabadi and Sasannejad [16] used 10% PVI for 10 minutes for toothbrush disinfection and noted an antibacterial effect similar to that of chlorhexidine disinfectants. Similarly, in our study, there was a significant reduction in the bacterial CFU in group VI (10% PVI, 73.43%). This suggests that PVI disinfectants exhibit an antibacterial effect on contaminated toothbrushes. Thus, similar to chlorhexidine, PVI solution can be recommended as a suitable disinfectant for toothbrushes.

Microwave radiation is an effective way to sterilize acrylic resins. The antibacterial effect of microwave irradiation for 6-10 minutes was demonstrated on a removable denture infected with *S. epidermidis*, *S. aureus*, *K. pneumoniae*, *B. subtilis*, and *Candida albicans* [15]. Another study reported that using a microwave at high power for five minutes to sterilize toothbrushes infected with *S. mutans* was adequate; however, this method failed to eliminate all microorganisms. In addition, the toothbrush was destroyed after five minutes of irradiation [17]. This study found that 1% sodium hypochlorite significantly reduced the number of all microorganisms detected and eliminated almost all streptococci; the same concentration was also influential in eliminating *E. coli*. This result is consistent with that of a* previous study *that demonstrated the antibacterial efficiency of 1% sodium hypochlorite, 2% chlorhexidine digluconate, and 50% vinegar. The systematic use of these compounds for prosthesis disinfection in dentistry likely contributes to enhanced infection control and reduces the danger of cross-contamination [14].

UV light can inactivate microorganisms by breaking chemical bonds containing DNA atoms. In this study, exposing toothbrush samples to UV light for seven minutes resulted in 76.91% bacterial count reduction. Prolonged exposure to UV radiation can completely inactivate microorganisms with various UV disinfectants that have recently become available [18]. The effectiveness of UV sanitization devices against bacteria and viruses has also been studied. VIOlight and HIGHDENT have been shown to reduce the number of Gram-negative and Gram-positive bacteria by 83% and 100%, respectively [19]. However, the use of DenTek UV for 10 minutes was not effective against *S. mutans*. Although prolonged exposure to UV light can eliminate more bacteria, the equipment automatically shuts down after 10 minutes [17]. The UV toothbrush holder used in this study was the most expensive among all other toothbrush disinfection kits used. Thus, the cost-effectiveness of UV toothbrush holders should be investigated.

Although white vinegar is not widely used for disinfecting teeth because of its toxicity, it is a promising alternative to disinfectants against different bacterial strains [20]. However, only a few studies on the use of white vinegar in dentistry have been reported. White vinegar is commonly used to disinfect toothbrushes and acrylic paints at concentrations of 50% and 100%, respectively. Acetic acid 100% (white vinegar) exhibited excellent antibacterial activities against *C. albicans* and *S. aureus* on acrylic resins [13]. It was also as effective as 1% sodium hypochlorite and 2% chlorhexidine digluconate solutions against *C. albicans*, *E. coli*, and *S. mutans*. Komiyama et al. found that 50% white vinegar was effective against *S. aureus*, *S. mutans*, and *S. pyogenes* but not against *C. albicans* [21]. Immersion of a toothbrush in 50% and 100% white vinegar for 10 minutes was very effective in eliminating all bacteria and was the most effective treatment method against *S. mutans* and *S. aureus* [22].

Abd-Ulnabi* *[7]* *detected *Pseudomonas*, *S. aureus*, *S. epidermidis*, and yeast colonies on the toothbrushes of healthy individuals, similar to our findings. However, this study did not evaluate the samples for yeast. Bhat et al. reported that *S. mutans* were the most commonly found species on contaminated toothbrush samples cultured on Mitis Salivarius agar plates, which is also similar to our findings [23]. Another study noted the following predominant bacterial species from contaminated toothbrushes: *Bordetella* spp., *Salmonella*, *Candida*, *Klebsiella*, *Proteus*, *Citrobacter*, *Pseudomonas* spp., *S. aureus*, *Providencia*, *Lactobacillus*, *Chromobacterium*, *B. cereus*, enterococci, and non-hemolytic streptococci [24]. Only half of these species were identified in this study.

## Conclusions

We evaluated the prevalence of different microorganisms on used toothbrushes and studied the effectiveness of various disinfection methods. A significant reduction in the bacterial count was observed in all disinfection methods, with 2% glutaraldehyde and 3% hydrogen peroxide showing the most effective mean decrease in bacterial count. Toothbrushes are recommended to be replaced every three months; moreover, sick children and adults should frequently change their toothbrushes to prevent reinfection or spreading infection.
